# PRL-3 and MMP9 Expression and Epithelial-Mesenchymal Transition Markers in Circulating Tumor Cells From Patients With Colorectal Cancer: Potential Value in Clinical Practice

**DOI:** 10.3389/fonc.2022.878639

**Published:** 2022-04-29

**Authors:** Xiao-Cui Hong, Qi-Lian Liang, Man Chen, Hai-Xia Yang, Jie Huang, Si-Lin Yi, Zhen-Wei Wang, Hai-Yan Liang, Ding-Yue Zhang, Zeng-Yi Huang

**Affiliations:** ^1^ Oncology Center, Affiliated Hospital of Guangdong Medical University, Zhanjiang, China; ^2^ Pathology Department, Affiliated Hospital of Guangdong Medical University, Zhanjiang, China

**Keywords:** circulating tumor cells (CTCs), colorectal cancer (CRC), PRL-3, MMP9, epithelial-mesenchymal transition (EMT)

## Abstract

**Objective:**

To evaluate the clinical correlation of epithelial-mesenchymal transition (EMT) with PRL-3 and MMP9 expression in the circulating tumor cells (CTCs) of patients with colorectal cancer (CRC).

**Materials and Methods:**

Between January 2016 and December 2018, the EMT phenotype-based subsets of CTCs and the expression levels of PRL-3 and MMP9 in CTCs were identified, and their clinical values in 172 patients were evaluated. The CTCs were isolated, classified, and counted using the CanPatrol™ CTC filtration system. The CTC subsets (epithelial cells, mesenchymal cells and biphenotypic cells), as well as PRL-3 and MMP9 expression, were detected by RNA *in situ* hybridization.

**Results:**

CTCs were detected in 93.0% (160/172) of the included patients with CRC. Positive PRL-3 and MMP9 expression in CTC and M-CTC was found in 75.0% (102/136) and 80.8% (97/120) of the patients, respectively. The proportion of patients with positive PRL-3 and MMP9 expression in M-CTC was significantly associated with distant metastasis (p<0.05). The patients with ≥6 CTCs tended to show poorer progression-free survival (PFS) and overall survival (OS) rates (p=0.016, 0.02, respectively), and the patients with ≥3 M-CTC also showed poor PFS (p=0.0013). Additionally, the patients with positive PRL-3 and MMP9 expression in CTCs had significantly poorer PFS (p=0.0024) and OS (p=0.095) than the patients with negative PRL-3 and MMP9 expression. Multivariate Cox analysis uncovered that positive PRL-3 and MMP9 expression in CTCs may be an independent prognostic factor for worse PFS.

**Conclusion:**

EMT phenotypes and CTC numbers can be used as prognostic indicators for metastasis and survival in patients with CRC, and the combination of PRL-3 and MMP9 expression in CTCs is a promising clinical marker for patients with CRC.

## Background

Colorectal cancer (CRC) is the third most common fatal cancer worldwide. Similar to other cancers, patients with CRC diagnosed at earlier or intermediate stages have better prognoses than those at advanced stages (1, 2). In China, the most populous country, more than a third of patients are diagnosed with CRC when their tumors are advanced (3). Despite improved detection and clinical treatment, the high incidence of recurrence and metastasis makes the prognosis of CRC still not optimistic. All of these lead to low survival rates. Therefore, there is an urgent need for new methods to detect early CRC and to continuously monitor the response to antitumor therapy, so as to reduce the risk of disease progression and metastasis.

Circulating tumor cells (CTCs), which enter the circulatory system from primary or metastatic tumors, have been found in a variety of tumors; CTC distribution plays a vital role in cancer progression (4–6). The clinical relevance of CTCs as prognostic indicators of treatment response has been demonstrated in several cancer types (7–10). CTCs can be defined as epithelial type (E-CTC), mesenchymal type (M-CTC), and biphenotypic epithelial/mesenchymal type (B-CTC) using different expression models of epithelial and mesenchymal markers (11). Recent studies have shown that the epithelial-mesenchymal transition (EMT) phenotype of CTCs may promote tumor metastasis. In the whole process of hematogenous metastasis, CTCs undergo EMT from E-CTC to M-CTC to better penetrate the basement membrane, cross the blood vessel wall, and reach the blood circulation to form metastatic foci (12–15). However, the expression and function of many genes in CTCs have not been confirmed in CRC. Identifying and discovering new biomarkers from CTCs are essential and promising, because they can provide new ideas for clinical research and treatment.

Phosphatase of regenerating liver-3 (PRL-3) belongs to the protein tyrosine phosphatase (PTP) family and is an interesting metastasis gene first identified in CRC (16, 17). Over the past decade, several pieces of evidence implicated its role in the metastatic progression of multiple cancer types (18–20). PRL-3 promotes the invasion and metastasis of tumor cells by promoting EMT, and this mechanism has been confirmed in CRC (21–23). PRL-3 overexpression is found in nearly all metastatic lesions derived from CRC but at lower levels in the corresponding primary tumors and normal epithelium (24). Therefore, we suggest that PRL-3 expression may be a biomarker associated with poor prognosis in CRC.

Several studies have also highlighted that PRL-3 facilitates tumor cell invasiveness by regulating extracellular matrix degradation, which is mediated by matrix metalloproteinases (MMPs) (25). In particular, PRL-3 promotes tumor cell invasion by upregulating MMP-7 in human CRC (26). Likewise, MMP-2 and MMP-9 expression levels are downregulated by PRL-3 siRNA in CRC cells (27). Among more than 20 MMPs, matrilysin (MMP-9) appears to be one of the most important MMPs in CRC, because it is closely related with tumor invasion and metastasis in CRC (28–30). PRL-3 and MMP9 jointly affect tumor metastasis; hence, the combined detection of their expression in CRC can more comprehensively evaluate tumor metastasis potential.

Currently, more and more new genes have been found and vertified in the CTCs of patients with CRC, and their expression has been discovered to play an important role in CRC development. Nevertheless, the clinical implications of combined PRL-3 and MMP-9 expression in the CTCs of patients with CRC have not been demonstrated. In this study, we used CanPatrol™ system to characterize CTC phenotype in the peripheral blood samples of patients with CRC and detect the expression of PRL-3 and MMP-9 in CTCs by mRNA *in situ* hybridization (RNA‐ISH) method. The objective of our study was to investigative the functional role of PRL-3 and MMP-9 in CTCs, uncover the correlation of PRL-3 and MMP-9 expression in CTC subgroups with the most common clinical variables of patients with CRC, and assess the potential clinical value of CTCs in CRC.

## Materials and Methods

### Patients and Samples

This study was registered in the Chinese Clinical Trial Registry (Identifier ChiCTR-RDD-16007819) and approved by the Ethics Committee of the Affiliated Hospital of Guangdong Medical University (PJ2015117KT). Written informed consents were provided by all the patients.

A total of 172 patients with CRC were enrolled from the Affiliated Hospital of Guangdong Medical University from January 2016 to December 2018. The selected patients met the following criteria: (a) had a pathologically diagnosed CRC, (b) did not receive any antitumor treatment before surgery, and (c) had no other complications or previous malignancies. The exclusion criteria were as follows: (a) primary malignant tumor of other tissues before or during the study and (b) poor compliance to follow-up.

Follow-up was carried out completely. Patients were followed up every 3 months for the first 2 years and every 6 months thereafter. Routine examinations for follow-up included physical examination, abdominal ultrasound, magnetic resonance imaging and chest CT, routine blood examination during clinical examination and optional PET-CT scanning for signs of tumor recurrence or metastasis. Progression free survival (PFS) was defined as the first progression date or the last follow-up date from the date of diagnosis in any site, and overall survival (OS) was defined as the period from the date of diagnosis to the date of death or the last follow-up date.

### Isolation of CTCs and Detection of PRL-3 and MMP9 Expression

The CanPatrol™ CTC filtration system (31) was used to isolatd, classify, and count the CTCs. The filtration system included a membrane with 8 μm-diameter calibrated pores (Sur Exam, Guangzhou, China), a manifold vacuum plate with valve settings (Millipore Billerica, Ma, USA), an E-Z 96 vacuum manifold (Omega, Norcross, GA, USA), and a vacuum pump (Auto Science, Tianjin, China). Peripheral blood samples (5 mL) of the patients were collected into EDTA tubes and stored at 4°C within 4 h. Before filtration, the red blood cells were removed by erythrolysis and resuspended with PBS containing 4% formaldehyde for 5 min. The cell suspension was transferred to a filter tube and pumped at a pressure of at least 0.08 MPa to collect the CTCs separated onto the membrane.

According to different expression models of EMT markers (epithelial biomarkers [EpCAM], mesenchymal biomarkers [Vimentin], biophenotypic biomarkers [CD45]) and target markers PRL-3 and MMP9 in CTCs, the CTCs were classified and counted by the RNA‐ISH method (31, 32). Then, the cells were stained with DAPI and analyzed with an automatic imaging fluorescence microscope. The red, green, and purple fluorescent signal points represent the expression of epithelial genes, mesenchymal genes, and the *PRL-3 and MMP9* genes in CTCs, respectively.

### Statistical Analysis

The correlation between CTCs and clinical variables was analyzed by chi square test. Kaplan–Meier analysis and log rank test were used to generate survival curves. Univariate and multivariate Cox analyses were performed and adjusted hazard ratios (HRs) were obtained to evaluate the independent prognostic factors. All data were analyzed using the SPSS 25.0 software. P < 0.05 was considered statistically significant.

## Results

### Clinicopathological Features of the Patients and CTCs

**Figure 1:**
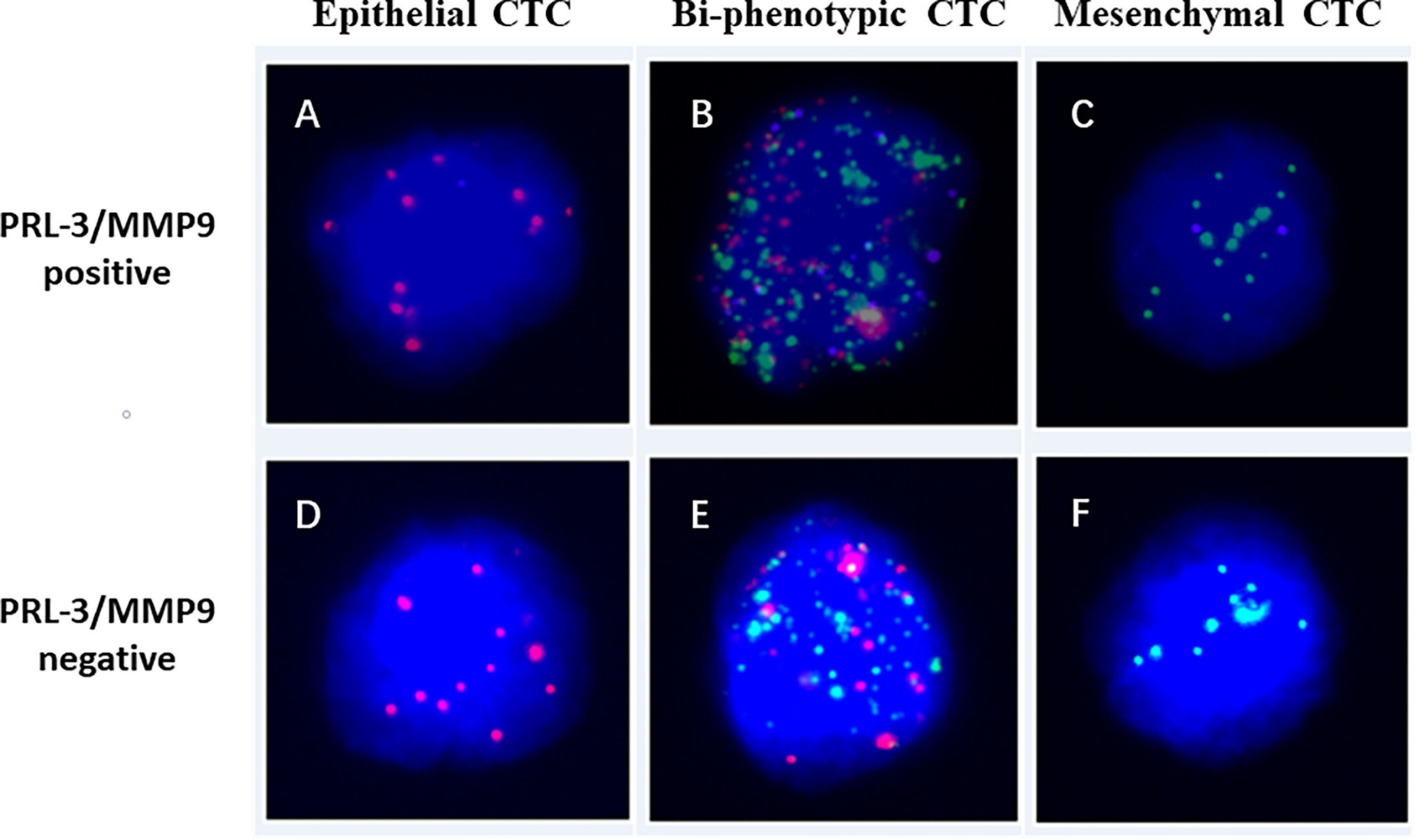
Images of PRL-3 and MMP9 positive **(A–C)** or negative **(D–F)** expression in three subgroups of CTCs from patients with colorectal cancers, based on RNA *in situ* hybridization staining of epithelial (**A, D**: red dots), mesenchymal (**B, E**: green dots), and biophenotypic CTCs (**C, F**: red and green dots). PRL-3, phosphatase of regenerating liver-3; MMP9, matrix metalloproteinase-9; CTCs, circulating tumor cells.

A total of 172 patients with were included in this study. The detailed clinicopathological features of the patients are shown in **Table 1**. CTCs (≥1 per 5 mL of blood) were detected in 160 of the 172 patients (93.0%) with a median count of 6.0 CTCs (range 0–115/5 mL blood). **Figure 1** shows the CTC subgroups isolated from the CRC cells of patients by RNA-ISH based on the staining of epithelial and mesenchymal markers (**Figure 1D** for E-CTC, **Figure 1E** for B-CTC, and **Figure 1F** for M-CTC). In total, ≥3 CTCs/5 mL were detected in 136 of 172 patients (79.1%). These patients were considered CTC positive. In addition, ≥1 M-CTC/5 mL was found in 120 enrolled patients. These patients were considered M-CTC positive.

**Table 1:** Univariate analyses between CTCs and clinicopathological factors (n=172).

Variables	N (%)	CTCs		P	M-CTC		P
		≥3	<3		≥1	0	
All patients	172 (100.0)	136 (79.1)	36 (20.9)		120 (69.8)	52 (30.2)	
Gender							
Male	107 (65.1)	84 (78.5)	23 (24.5)	0.815	77 (69.6)	30 (30.4)	0.421
Female	65 (34.9)	52 (80.0)	13 (20.0)		43 (70.0)	22 (30.0)	
Depth of invasion							
T1–T3	118 (68.6)	90 (76.3)	28 (23.7)	0.182	82 (69.5)	36 (30.5)	0.907
T4	54 (31.4)	46 (85.2)	8 (14.8)		38 (70.4)	16 (29.6)	
Lymphatic metastasis							
No	82 (47.7)	64 (78.0)	18 (22.0)	0.753	52 (63.4)	30 (36.6)	0.083
Yes	90 (52.3)	72 (80.0)	18 (20.0)		68 (75.6)	22 (24.4)	
Distant metastasis							
No	128 (74.4)	101 (78.9)	27 (21.1)	0.928	88 (68.8)	40 (31.2)	0.620
Yes	44 (25.6)	35 (79.5)	9 (20.5)		32 (72.7)	12 (27.3)	
TNM stage							
I+II	65 (37.8)	49 (75.4)	16 (24.6)	0.354	41 (63.1)	24 (36.9)	0.136
III+IV	107 (62.2)	87 (81.3)	20 (18.7)		79 (73.8)	28 (26.2)	

### Associations Between CTC Phenotypes and Patient Clinicopathological Features

The clinicopathological features of CRC, including gender, depth of invasion, lymph node metastasis, distant metastasis, and TNM stage were analyzed to explore the clinical relevance of CTC phenotypes. Notably, the total number of CTCs and M-CTCs detected from the patients had no significant correlation with any of the assessed clinicopathological parameters (P>0.05, **Table 1**). Although not statistically significant (P=0.084), M-CTC was positively correlated with lymph node metastasis, in which the rates of M-CTC-positive patients with and without lymphatic metastasis were 75.6% and 63.4%, respectively (**Table 1**). These data indicate that the increase in the number of M-CTC can reflect the increase in metastasis risk to some extent.

### Associations Between the Expression of PRL-3 and MMP9 in CTCs and Patient Clinicopathological Features

A total of 160 patients met the criteria (≥1 CTCs/5 mL blood) and were included for PRL-3 and MMP9 analyses. The expression images of PRL-3 and MMP9 in the three subgroups of CTCs are shown in **Figures 1A–C**. The associations between the expression of PRL-3 and MMP9 (in CTCs and M-CTCs) and the clinicopathological features of the patients with CRC are listed in **Table 2**. Positive PRL-3 and MMP9 expression in CTCs was found in 75.0% (102/136) of patients, and its expression in CTCs exhibited no significant association with any clinicopathological feature (P>0.05, **Table 2**). Although the difference did not reach statistical significance (P= 0.089), those with positive expression had a higher rate of metastasis (71.3% vs 85.7%) compared with the patients with negative PRL-3 and MMP9 expression in CTCs. The data imply that the combination of PRL-3 and MMP9 expression in CTC subgroups can improve the clinical prediction of CRC metastasis.

**Table 2:** Demographics of patients with CTCs≥3/5 mL blood (n=136) and mCTC≥1/5 mL blood (n=120) used for analysis of PRL-3 and MMP9 expression and its association with clinicopathologic features.

	PRL-3 and MMP9 expression in CTCs		P value	PRL-3 and MMP9 expression in MCTC		P value
Characteristics	Negative	Positive		Negative	Positive	
Gender						
Male	23	61	0.415	16	61	0.548
Female	11	41		7	36	
Depth of invasion						
T1–T3	23	67	0.834	15	67	0.721
T4	11	35		8	30	
Lymphatic metastasis						
No	18	46	0.427	11	41	0.629
Yes	16	56		12	56	
Distant metastasis						
No	29	72	0.089	21	67	0.030
Yes	5	30		2	30	
TNM stage						
I+II	16	33	0.122	11	20	0.070
III+IV	18	69		12	77	

Previous studies on EMT mechanism in CTC suggested that the proportions of M-CTC and E-CTC could reflect metastasis risk. In our study, positive PRL-3 and MMP9 expression in M-CTC, E-CTC, and B-CTC was found in 80.8% (97/120), 52.6% (41/78), and 86.7% (124/143) of patients, respectively. PRL-3 and MMP9 expression levels were remarkably higher in M-CTC than in E-CTC, suggesting that PRL-3 and MMP9 may be associated with the degree of malignancy and promote metastasis.

The relationship between the expression of PRL-3 and MMP9 and the characteristics of patients with CRC supports this hypothesis. The patients with positive PRL-3 and MMP9 expression in M-CTC accounted for 93.75% of patients with metastasis and 76.1% of patients without metastasis; this finding demonstrates a significant correlation between the two variables (P=0.030, **Table 2**). The result showed that the PRL-3 and MMP9 expression in M-CTC is closely related to metastasis. In addition, the presence of M-CTC was clearly correlated with TNM stage but without statistical significance. In the more aggressive disease state, the proportion of M-CTC was substantially higher, which suggests that M-CTC may be a new alternative marker for tumor invasion.

### Associations Between the Expression of PRL-3 and MMP9 and Survival

**Figure 2:**
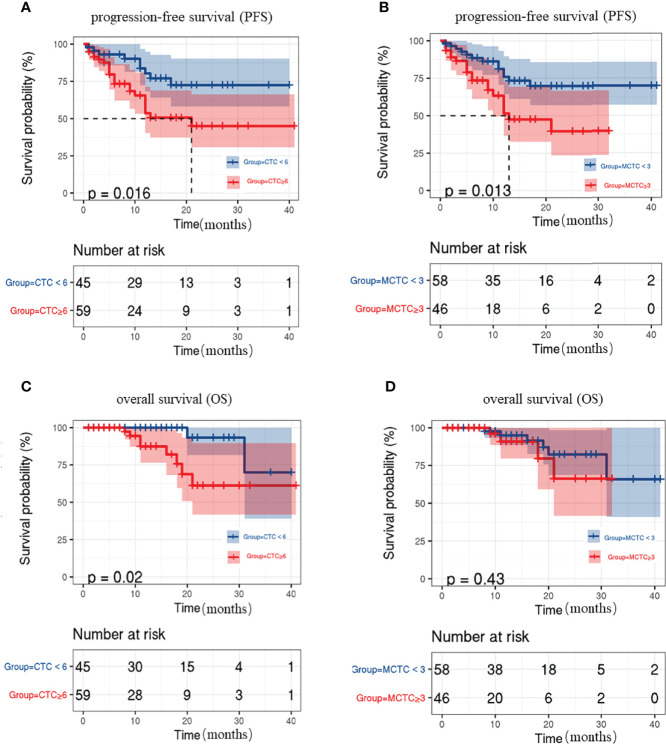
Kaplan-Meier curves for progression-free survival (PFS) and overall survival (OS) in CRC patients. **(A, C)** relation between CTC number and **(A)**PFS and **(C)**OS rate. **(B, D)** relation between M-CTC number and **(B)** PFS and **(D)** OS rate.

A total of 104 patients were followed up for disease assessment for a period of 1–41 months and with a median follow-up of 11 months. Previous studies on CRC uncovered that the number of CTC is associated with poor prognosis. Thus, we evaluated the associations between CTC status and survival during follow-up. The patients with ≥6 CTCs showed poorer PFS and OS rates than those without CTCs (P=0.016, 0.02, respectively; **Figures 2A, C**). The patients with ≥3 M-CTCs had poorer PFS rates (P=0.0013, **Figure 2B**) but had no remarkable difference in OS (P>0.05, **Figure 2D**) than those without M-CTCs.

**Figure 3:**
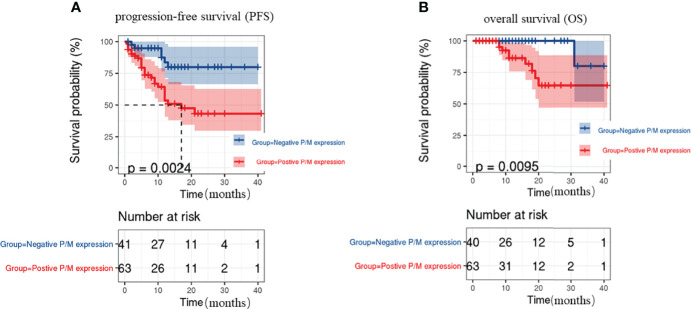
Kaplan–Meier’s survival curves for **(A)** PFS of CRC patients with or without PRL-3 and MMP9 positive expression on CTCs, **(B)** OS of CRC patients with or without PRL-3 and MMP9 positive expression on CTCs.

PFS and OS were analyzed by considering the CTC status, as well as PRL-3 and MMP9 expression. The survival curves revealed that the patients with positive PRL-3 and MMP9 expression in CTCs had significantly poorer PFS (P=0.0024, **Figure 3A**) and OS (P=0.0095, **Figure 3B**) than patients with negative PRL-3 and MMP9 expression. The univariate and multivariate analyses for OS and PFS are presented in **Table 3**, respectively. The univariate analysis of PFS revealed that ≥6 total CTCs (P=0.022), ≥3 M-CTC (P=0.018), and positive PRL-3 and MMP9 expression in CTCs were prognostic indicators. Multivariate analysis further revealed that only the PRL-3 and MMP9 expression in CTCs served an independent prognostic indicator for PFS in patients with CRC (P=0.042, **Table 3**). PRL-3 and MMP9 expression in CTCs was a prognostic factor in the univariate analysis of OS but not in the multivariate analysis of OS.

**Table 3:** Univariate and multivariate Cox regression analyses of associations between CTCs, PRL-3 and MMP9 expression and patient survival.

	Univariate analysis			Multivariate analysis		
	P value	HR	95% CI	P value	HR	95% CI
Associated with PFS						
CTCs (≥6 vs <6)	0.022	2.473	1.140-5.363	0.814	0.861	0.247-2.999
M-CTC (≥3 vs <3)	0.018	2.357	1.159-4.792	0.314	1.679	0.612-4.605
P/M expression (Negative vs Positive)	0.005	3.573	1.467-8.700	0.042	3.106	1.041-9.272
Associated with OS						
CTCs (≥6 vs <6)	0.138	0.717	0.717-10.979	0.721	1.358	0.919-7.291
M-CTC (≥3 vs <3)	0.970	0.247	0.247-3.828	0.341	0.471	0.065-2.218
P/M expression (Negative vs Positive)	0.030	10.549	1.250-89.025	0.053	10.110	0.973-105.090

## Discussion

With the continuous deepening of related CTC research and the development of detection technology, the monitoring of CTCs in the peripheral blood of patients with cancer has become an important means of tumor precision medicine (5, 6, 33). Compared with traditional biopsy techniques, CTC detection has low trauma, repeated sampling, and real-time “update and feedback” of tumor load; thus, it has become a hot spot in the oncology research field. In recent years, CTC phenotypes have been mentioned in an increasing number of studies on EMT, as they seem to be related to the process of metastasis (34, 35). This notion was consistent with our findings in CRC.

Even a small tumor can shed millions of cancer cells. However, metastasis does not occur immediately in all patients with cancer, and it often occurs after a long incubation period. We found that the presence of CTC could be detected in the peripheral blood of patients with early and late CRC, which indicates that CTC has existed before the occurrence of metastasis and could also explain why some patients with early cancer progressed so rapidly.

The CanPatrol™ system was used in this study. The detection technique was based on the separation and enrichment of CTCs in the peripheral blood by immune removal combined with nanofiltration. The CTCs were divided into three types according to the expression of epithelial–interstitial markers. Studies have shown that EMT plays a key role in the formation of CTC phenotypes (11, 14, 15). EMT is a biological phenomenon, in which the expression of epithelial markers is downregulated and the expression of interstitial markers is upregulated; EMT changes the morphology, physical properties, and cytoskeleton of tumor cells, which result in stronger tumor cell invasion, migration, and viability *in vivo (* 12, 36). As expected, we did find that the number of M-CTC tended to be more associated with CRC metastasis than the other two CTC phenotypes. Our findings suggest that EMT is involved in the process of CRC metastasis. It does not mean that all patients with mesenchymal marker-positive CTCs will have increased metastasis risk. In addition, the study of biphenotypic (hybrid phenotype) also deserves our attention. In the past, it was often regarded as the intermediate stage of the transition from epithelial phenotype to mesenchymal phenotype, but now it is also predicted to be related to some specific biological behaviors of tumors, which is left blank in our study.

Previous researches have shown that the number of CTCs may be a risk factor for CRC progression and survival (9, 37–39). Consistent with previous research, our data show the presence of ≥6 CTCs or ≥3 M-CTC made the patients shown a poorer PFS than those without CTCs or M-CTCs. However, the patients with ≥3 M-CTC had poorer PFS than the patients with <3 M-CTC but had no correlation with OS. These data revealed the relevance between the number of total CTCs and M-CTCs and the survival and prognosis of patients with CRC. In the subsequent multivariate analysis, which included other potential prognostic factors, the statistical data did not demonstrate that total CTC and/or M-CTC numbers are independent predictors of CRC prognosis. This result suggests that a high CTC and/or M-CTC count does not mean poor survival and prognosis. CTC has high heterogeneity and mutation probability; hence, analysis through EMT markers alone is not enough to represent the characteristics of CTC. Therefore, the expression of other genes was analyzed in total CTCs and CTC subgroups.

PRL-3 belongs to the PTP family, whereas MMP9 is a member of the MMP family; PRL-3 and MMP9 play key roles in promoting CRC cell invasion, metastasis, and EMT (20). Furthermore, several studies also revealed that the role of PRL-3 in metastasis is mediated by MMPs, and the combined detection of the expression of PRL-3 and MMP9 in CRC can more comprehensively evaluate tumor metastasis potential than using PRL-3 or MMP9 expression alone (25–27). Consistent with previous research, we found that positive PRL-3 and MMP9 expression in M-CTC was associated with distant metastasis. This result suggests that PRL-3 and MMP9 are engaged in this process. Therefore, the expression of PRL-3 and MMP9 in CTCs could be applied to forecast and evaluate metastasis in patients with CRC. Next, the PFS and OS between CTC status and PRL-3 and MMP9 expression were analyzed. The survival curves revealed that the patients with positive PRL-3 and MMP9 expression in CTCs had remarkably poorer PFS and OS than patients with negative PRL-3 and MMP9 expression. The univariate and multivariate Cox analyses uncovered that PRL-3 and MMP9 expression in CTCs served as an independent prognostic indicator for the PFS of patients with CRC but not for their OS.

However, the data of single-center study and the shallow study of hybrid phenotype are the potential limitation of our study. In previous studies, the individual phenotype of CTC (especially mesenchymal phenotype) has been confirmed to be associated with lymph node metastasis, high-grade tumor germination and shorter OS, which is also consistent with our results. But the most challenging cases remain the hybrid CRCs, whose particular behavior should be examined on a molecular level. Unfortunately, we are unable to draw an accurate conclusion about its role at present. Although this study is a prospective study with a large sample size involving patients with CRC, caution should be exercised in drawing any conclusions, and more detailed subgroup analysis and more in-depth molecular research should be performed in subsequent studies. Further multicenter studies are needed to confirm our findings.

## Conclusions

This study suggests that the expression of PRL-3 and MMP9 in CTCs is a promising prognostic marker associated with PFS in patients with CRC. The results of our study need to be verified by multicenter studies, and the value of PRL-3 and MMP9 in improving the survival of patients with CRC should be further evaluated in combination with clinical diagnostic and treatment strategies.

## Data Availability Statement

The original contributions presented in the study are included in the article/supplementary materials. Further inquiries can be directed to the corresponding author.

## Ethics Statement

All procedures carried out in studies involving human participants were in accordance with the ethical standards of the Ethics Committee at the Affiliated Hospital of Guangdong Medical University (PJ2015117KT) and with the 1964 Helsinki declaration and its subsequent revisions or similar ethical standards. The patients/participants provided their written informed consent to participate in this study.

## Author Contributions

Conceptualization, X-CH and Q-LL. Formal analysis, MC and H-XY. Investigation, JH and Z-WW. Methodology, S-LY, and H-YL. Resources, D-YZ, and Z-YH. Writing–original draft, X-CH. Writing–review and editing, Q-LL. All authors contributed to the article and approved the submitted version.

## Funding

Supported by the Key Research Platforms and Projects of Universities (Characteristic innovation projects) in Guangdong Province, China (No. 2018KTSCX077), the Technology Planning Project of Guangdong Province, China (No. 2014A020212291), the Science and Technology Development Special Fund Competitive Allocation Project of Zhanjiang, China (No. 2018A01028) and the Affiliated Hospital of Guangdong Medical University Clinical Research Program, China (No. LCYJ2018A005).

## Conflict of Interest

The authors declare that the research was conducted in the absence of any commercial or financial relationships that could be construed as a potential conflict of interest.

## Publisher’s Note

All claims expressed in this article are solely those of the authors and do not necessarily represent those of their affiliated organizations, or those of the publisher, the editors and the reviewers. Any product that may be evaluated in this article, or claim that may be made by its manufacturer, is not guaranteed or endorsed by the publisher.

## References

[1] Siegel RL Miller KD Jemal A. Cancer Statistics, 2020. CA Cancer J Clin (2020) 70(1):7–30. doi: 10.3322/caac.21590 31912902

[2] Siegel RL Miller KD Goding Sauer A Fedewa SA Butterly KLF Anderson JC. Colorectal Cancer Statistics, 2020. CA Cancer J Clin (2020) 70(3):145–64. doi: 10.3322/caac.21601 32133645

[3] Zheng RS Sun KX Zhang SW Zeng HM Zou XN Chen R. Analysis on the Prevalence of Malignant Tumors in China in 2015. Chin J Oncol (2019) 41(1):19–28. doi: 10.3760/cma.j.issn.0253-3766.2019.01.005 30678413

[4] Alix-Panabières C Pantel K. Clinical Applications of Circulating Tumor Cells and Circulating Tumor DNA as Liquid Biopsy. Cancer Discov (2016) 6(5):479–91. doi: 10.1158/2159-8290.CD-15-1483 26969689

[5] Ignatiadis M Lee M Jeffrey SS. Circulating Tumor Cells and Circulating Tumor DNA: Challenges and Opportunities on the Path to Clinical Utility. Clin Cancer Res (2015) 21(21):4786–800. doi: 10.1158/1078-0432.CCR-14-1190 26527805

[6] Alix-Panabières C Pantel K. Challenges in Circulating Tumour Cell Research. Nat Rev Cancer (2014) 14(9):623–31. doi: 10.1038/nrc3820 25154812

[7] Dasgupta A Lim AR Ghajar CM. Circulating and Disseminated Tumor Cells: Harbingers or Initiators of Metastasis. Mol Oncol (2017) 11(1):40–61. doi: 10.1002/1878-0261.12022 28085223 PMC5423226

[8] Liu DG Xue L Li J Yang Q Peng JZ. Epithelial-Mesenchymal Transition and GALC Expression of Circulating Tumor Cells Indicate Metastasis and Poor Prognosis in non-Small Cell Lung Cancer. Cancer Biomark (2018) 22(3):417–26. doi: 10.3233/CBM-170995 PMC13078463 29758927

[9] Wang WY Wan L Wu SY Yang JG Zhou Y Liu F. Mesenchymal Marker and LGR5 Expression Levels in Circulating Tumor Cells Correlate With Colorectal Cancer Prognosis. Cell Oncol (Dordr) (2018) 41(5):495–504. doi: 10.1007/s13402-018-0386-4 29949050 PMC12995228

[10] Li YJ Luo Y Xie XQ Li P Wang F. The Prognostic Value of COX-2 Expression on Circulating Tumor Cells in Nasopharyngeal Carcinoma: A Prospective Analysis. Radiother Oncol (2018) 129(2):396–402. doi: 10.1016/j.radonc.2018.07.022 30082142

[11] Wu S Liu S Liu Z Huang JF Pu XY Li J. Classification of Circulating Tumor Cells by Epithelial-Mesenchymal Transition Markers. PloS One (2015) 10(4):e0123976. doi: 10.1371/journal.pone.0123976 25909322 PMC4409386

[12] Pastushenko I Blanpain C. EMT Transition States During Tumor Progression and Metastasis. Trends Cell Biol (2019) 29(3):212–26. doi: 10.1016/j.tcb.2018.12.001 30594349

[13] Cao H Xu E Liu H Wan LD Lai MD. Epithelial-Mesenchymal Transition in Colorectal Cancer Metastasis: A System Review. Pathol Res Pract (2015) 211(8):557–69. doi: 10.1016/j.prp.2015.05.010 26092594

[14] Javaid S Zhang J Smolen GA Yu M Wittner BS Singh A. MAPK7 Regulates EMT Features and Modulates the Generation of CTCs. Mol Cancer Res (2015) 13(5):934–43. doi: 10.1158/1541-7786.MCR-14-0604 PMC4433453 25678598

[15] Kölbl AC Jeschke U Andergassen U. The Significance of Epithelial-To-Mesenchymal Transition for Circulating Tumor Cells. Int J Mol Sci (2016) 17(8):1308. doi: 10.3390/ijms17081308 PMC5000705 27529216

[16] Bardelli A Saha S Sager JA Romans KE Xin BZ Markowitz SD. PRL-3 Expression in Metastatic Cancers. Clin Cancer Res (2003) 9(15):5607–15. 14654542

[17] Duciel L Monraz Gomez LC Kondratova M Kuperstein I Saule S. The Phosphatase PRL-3 Is Involved in Key Steps of Cancer Metastasis. J Mol Biol (2019) 431(17):3056–67. doi: 10.1016/j.jmb.2019.06.008 31207239

[18] Xing X Lian S Hu Y Li ZY Zhang LH Wen XZ. Phosphatase of Regenerating Liver-3 (PRL-3) is Associated With Metastasis and Poor Prognosis in Gastric Carcinoma. J Transl Med (2013) 11:309. doi: 10.1186/1479-5876-11-309 24330843 PMC3878674

[19] Polato F Codegoni A Fruscio R Perego P Mangioni C Saha S. PRL-3 Phosphatase is Implicated in Ovarian Cancer Growth. Clin Cancer Res (2005) 11(19 Pt 1):6835–9. doi: 10.1158/1078-0432.CCR-04-2357 16203771

[20] Kato H Semba S Miskad UA Seo H Kasuga M Yokozaki H. High Expression of PRL-3 Promotes Cancer Cell Motility and Liver Metastasis in Human Colorectal Cancer: A Predictive Molecular Marker of Metachronous Liver and Lung Metastases. Clin Cancer Res (2004) 10(21):7318–28. doi: 10.1158/1078-0432.CCR-04-0485 15534108

[21] Al-Aidaroos AQ Yuen HF Guo K Zhang SD Chung TH Chng WJ. Metastasis-Associated PRL-3 Induces EGFR Activation and Addiction in Cancer Cells. J Clin Invest (2013) 123(8):3459–71. doi: 10.1172/JCI66824 PMC4011027 23867504

[22] Liu Y Zheng P Liu Y Ji H Liu XH Yao S. An Epigenetic Role for PRL-3 as a Regulator of H3K9 Methylation in Colorectal Cancer. Gut (2013) 62(4):571–81. doi: 10.1136/gutjnl-2011-301059 22345654

[23] Liu Y Zhou J Chen J Gao WZ Le Y Ding YQ. PRL-3 Promotes Epithelial Mesenchymal Transition by Regulating Cadherin Directly. Cancer Biol Ther (2009) 8(14):1352–9. doi: 10.4161/cbt.8.14.8695 19440036

[24] Peng L Ning J Meng L Shou C. The Association of the Expression Level of Protein Tyrosine Phosphatase PRL-3 Protein With Liver Metastasis and Prognosis of Patients With Colorectal Cancer. J Cancer Res Clin Oncol (2004) 130(9):521–6. doi: 10.1007/s00432-004-0563-x PMC12161869 15133662

[25] Kong L Li Q Wang L Sun TY. The Value and Correlation Between PRL-3 Expression and Matrix Metalloproteinase Activity and Expression in Human Gliomas. Neuropathology (2007) 27(6):516–21. doi: 10.1111/j.1440-1789.2007.00818.x 18021371

[26] Lee SK Han YM Yun J Lee CW Shin DS Ha YR. Phosphatase of Regenerating Liver-3 Promotes Migration and Invasion by Upregulating Matrix Metalloproteinases-7 in Human Colorectal Cancer Cells. Int J Cancer (2012) 131(3):E190–203. doi: 10.1002/ijc.27381 22131018

[27] Fan Y Zhang YL Zheng S. [Effects of Phosphatase of Regenerating Liver Cell-3 Gene Silence by RNA Interference on the Expression of Matrix Metalloproteinases-2,-9 in Human Colon Cancer Cells]. Zhonghua Wei Chang Wai Ke Za Zhi (2008) 11(5):477–81. doi: 10.3760/cma.j.issn.1671-0274.2008.05.019 18803055

[28] Walter L Pujada A Bhatnagar N Bialkowska AB Yang VW Laroui M. Epithelial Derived-Matrix Metalloproteinase (MMP9) Exhibits a Novel Defensive Role of Tumor Suppressor in Colitis Associated Cancer by Activating MMP9-Notch1-ARF-P53 Axis. Oncotarget (2017) 8(1):364–78. doi: 10.18632/oncotarget.13406 PMC5352126 27861153

[29] Wu MH Tzeng HE Wu CN Yueh TC Peng YC Tsai CH. Association of Matrix Metalloproteinase-9 Rs3918242 Promoter Genotypes With Colorectal Cancer Risk. Anticancer Res (2019) 39(12):6523–9. doi: 10.21873/anticanres.13867 31810917

[30] Bruno A Bassani B D'Urso DG Pitaku I Cassinotti E Pelosi G. Angiogenin and the MMP9-TIMP2 Axis Are Up-Regulated in Proangiogenic, Decidual NK-Like Cells From Patients With Colorectal Cancer. FASEB J (2018) 32(10):5365–77. doi: 10.1096/fj.201701103R 29763380

[31] Wu S Liu Z Liu S Li L Yang WW Xu JS. Enrichment and Enumeration of Circulating Tumor Cells by Efficient Depletion of Leukocyte Fractions. Clin Chem Lab Med (2015) 53(2):337. doi: 10.1515/cclm-2015-9999 25568985

[32] Qi LN Xiang BD Wu FX Ye JZ Zhong JH Wang YY. Circulating Tumor Cells Undergoing EMT Provide a Metric for Diagnosis and Prognosis of Patients With Hepatocellular Carcinoma. Cancer Res (2018) 78(16):4731–44. doi: 10.1158/0008-5472.CAN-17-2459 29915159

[33] Sun W Li G Wan J Zhu J Shen WQ Zhang Z. Circulating Tumor Cells: A Promising Marker of Predicting Tumor Response in Rectal Cancer Patients Receiving Neoadjuvant Chemo-Radiation Therapy. Oncotarget (2016) 7(43):69507–17. doi: 10.18632/oncotarget.10875 PMC5342494 27486758

[34] McInnes LM Jacobson N Redfern A Dowling A Thompson EW Saunders CM. Clinical Implications of Circulating Tumor Cells of Breast Cancer Patients: Role of Epithelial-Mesenchymal Plasticity. Front Oncol (2015) 5:42. doi: 10.3389/fonc.2015.00042 25767772 PMC4341429

[35] Książkiewicz M Markiewicz A Zaczek AJ. Epithelial-Mesenchymal Transition: A Hallmark in Metastasis Formation Linking Circulating Tumor Cells and Cancer Stem Cells. Pathobiology (2012) 79(4):195–208. doi: 10.1159/000337106 22488297

[36] Lamouille S Xu J Derynck R. Molecular Mechanisms of Epithelial-Mesenchymal Transition. Nat Rev Mol Cell Biol (2014) 15(3):178–96. doi: 10.1038/nrm3758 PMC4240281 24556840

[37] Groot Koerkamp B Rahbari NN Büchler MW Koch BH Weitz J. Circulating Tumor Cells and Prognosis of Patients With Resectable Colorectal Liver Metastases or Widespread Metastatic Colorectal Cancer: A Meta-Analysis. Ann Surg Oncol (2013) 20(7):2156–65. doi: 10.1245/s10434-013-2907-8 23456317

[38] Seeberg LT Waage A Brunborg C Hugenschmidt H Renolen A Stav I. Circulating Tumor Cells in Patients With Colorectal Liver Metastasis Predict Impaired Survival. Ann Surg (2015) 261(1):164–71. doi: 10.1097/SLA.0000000000000580 24509211

[39] Cai J Huang L Huang J Kang L Lin HC Huang PZ. Associations Between the Cyclooxygenase-2 Expression in Circulating Tumor Cells and the Clinicopathological Features of Patients With Colorectal Cancer. J Cell Biochem (2019) 120(4):4935–41. doi: 10.1002/jcb.27768 30260024

